# Approche de l’état podologique du patient diabétique hémodialysé chronique dans un centre hospitalier Marocain

**DOI:** 10.11604/pamj.2013.16.13.2289

**Published:** 2013-09-12

**Authors:** Nawal Benabdellah, Ilham Karimi, Yassamine Bentata, Intissar Haddiya

**Affiliations:** 1 tal Al farabi, Université Mohamed Premier, Faculté de médecine et de pharmacie, Oujda, Maroc

**Keywords:** Diabète, Etat podologique, hémodialysés chroniques, hypoparathyroidie, Hyperparathyroïdie secondaire, Anémie, infection, amputation, prise en charge multidisciplinaire, diabetes, podiatry state, chronic hemodialysis, hypoparathyroidism, secondary hyperparathyroidism, anemia, infection, amputation, multidisciplinary care

## Abstract

De plus en plus de patients diabétiques sont pris en charge en HDC. Les conséquences sont multiples, notamment les problèmes de pied diabétique qui demeurent mal connus du patient diabétique et des soignants ce qui retentit considérablement sur la qualité de prise en charge. Nous avons réalisé une Etude transversale en Novembre 2011 au centre d'hémodialyse de l'hôpital Al Farabi d'Oujda, incluant tous nos patients diabétiques HDC. Nous avons relevé les données démographiques, clinico-biologiques et dialytiques. Nous avons receuilli les éléments suivants: la présence ou non de douleur évalué par l’échelle visuelle analogique (EVA), le périmétre de marche, le recours ou non aux soins de pédicurie, le chaussage adapté, les antécédents de chirurgie vasculaire, l'existence ou non de troubles statiques et trophiques des pieds et la recherche d'une neuropathie par un test utilisant un brin de coton. L'analyse statistique est réalisée à l'aide du logiciel 11,5. Dans notre série, 12,9% des patients HDC sont diabétiques et présentent des troubles podologiques, aucun de nos patients n'a bénéficié de pontages ni angioplastie du membre inférieur alors que 8% ont subi des amputations. Le périmètre de marche est limité chez 75% des patients. 16,6% < 50 m, 16,6% < 100 m, 25% < 250 m, 16,6% ne marchent pas. Parmi, 58,3% présentent des troubles de la statique. 16,6% présentent des troubles trophiques. Quarante-cinq pour cent ont une neuropathie dépistée au test au brin de coton. Huit pour cent des patients portent des chaussures adaptées. Vingt-cinq pour cent des patients ont des plaies diverses. Les diabétiques en HDC représentent une population de patients fragiles qui nécessitent une prise en charge multidisciplinaire visant d'abord la prévention, le diagnostic précoce des ulcérations du pied diabétique, le traitement adapté aussi bien de l'infection que de l’équilibre glycémique, de nombreuses amputations chez ces patients peuvent être évitées grâce à un suivi et un programme d’éducation thérapeutique adapté.

## Introduction

Le diabète est une pathologie fréquente. Selon l'OMS, la prévalence du diabète va augmenter dans les 30 prochaines années de plus de 40% dans les pays industrialisés [1], le vieillissement de la population est un des facteurs explicatifs de cette épidémie [2, 3]. Au Maroc, les études concernant ce fléau sont peu nombreuses. L'enquête nationale sur les facteurs de risque cardiovasculaires menée en 2000 a montré que la prévalence du diabète est de 6,6% chez les personnes âgés de 20 ans et plus [4]. 25 à 30% des diabétiques type 2 développent une atteinte rénale, secondaire à une néphropathie diabétique dans la majorité des cas ou à une néphropathie vasculaire [5]. La néphropathie diabétique et la néphropathie vasculaire représentent plus de 50% des causes d'insuffisance rénale chronique terminale dans le monde [6]. Ainsi, de plus en plus de patients diabétiques sont pris en charge en hémodialyse chronique (HDC), la fréquence du diabète chez les nouveaux dialysés est de 41% en 2009 selon le registre rein 2009 [7].

Les patients diabétiques en hémodialyse chronique présentent de nombreuses complications, notamment le pied diabétique qui est responsable d'une grande morbidité et de graves incapacités, pèse lourdement sur la société, devenant un véritable problème de santé publique [8], cependant, ce problème demeure mal connu du patient diabétique et des soignants ce qui retentit considérablement sur la qualité de prise en charge. Le but de notre travail était de déterminer l’état podologique de nos patients diabétiques hémodialysés chroniques.

## Méthodes

Nous avons réalisés une étude transversale en Novembre 2011 au centre d'hémodialyse de l'hôpital Al Farabi d'Oujda, dans l'oriental marocain, incluant tous nos patients diabétiques HDC. Nous avons relevé chez nos patients les paramètres suivants:

**Démographiques:** à savoir l’âge, le sexe, le niveau d'instruction et les catégories socioprofessionnelles (1-Agriculteur exploitant 2-artisan commerçant ou chef d′entreprise 3-cadre profession intellectuelle supérieure, 4-profession intermédiaire, 5-employé, 6-ouvrier, 7-retraité, 8-inactif autre que retraité, 9-chômeur n′ayant jamais travaillé, 10-étudiant ou scolarisé).

**Cliniques:** Antécédents médico-chirurgicaux, les comorbidités (Hypertension artérielle (HTA) définie par une pression artérielle systolique> 140mmhg et/ou une pression artérielle diastolique > 90mmhg; Maladie cardio-vasculaire; néoplasie; Maladie de système évolutive), Index de masse corporelle (IMC) défini selon l'OMS: (Le surpoids correspond à un IMC égal ou supérieur à 25; de même que l'obésité correspond à un IMC égal ou supérieur à 30).

**Biologiques:** Hémoglobine (L'anémie défini par une baisse de l'hémoglobine (Hb) en dessous de 11 g/dl). Bbilan phospho-calcique (calcémie, phosphorémie, PTH intacte, 25OH vitamine D), bilan lipidique (cholestérol total, triglycérides), réserves alcalines.

**Dialytiques:** ancienneté en HDC, nombre de séance / semaine, la qualité de dialyse Kt/V(sp) Nos patients sont tous dialysé par des générateurs 4008 (Fresenius). Le Bain de dialyse utilisé est un bain standard, le tampon utilisé est le bicarbonate, avec une teneur en calcium à 1,75mmol/l, potassium à 2meq/ l, et Glucose à 0, 3g

**Thérapeutique:** les différents traitements en cours (traitement antihypertenseur, agents stimulants de l’érythropoïèse, supplémentation par le fer, calcium, chélateur de phosphore, analogues de la vitamine D).

**Caractéristiques podologiques:** la présence ou non de douleur évalué par l’échelle visuelle analogique (EVA), le périmètre de marche, le recours ou non aux soins de pédicurie, le chaussage adapté, les antécédents de chirurgie vasculaire, l'existence ou non de troubles statiques et trophiques des pieds, la recherche d'une neuropathie par un test utilisant un brin de coton.

L'analyse statistique a été réalisée par le logiciel SPSS 17.0. Les variables quantitatives sont exprimées en moyenne ± écart-type et les variables qualitatives en pourcentages. Une valeur p < 0,05 est considérée significative.

## Résultats

### Données démographiques, cliniques et dialytiques de nos patients diabétiques HDC (Tableau 1)

Notre centre compte 12 patients diabétiques, soit 12,9% de l'ensemble de nos patients HDC. L’âge moyen est de 53,2 ± 5,6 ans, il y a sept hommes et cinq femmes. Sur le plan socioprofessionnel, 7(58%) patients appartiennent à la catégorie 9, 3(25%) patients à la catégorie 8, alors que deux patients sont de la catégorie 10. Concernant le niveau d'instruction 10 patients sont analphabètes, tandis que 2 patients sont scolarisés, un avaient le niveau du primaire, et l'autre celui du secondaire. L'hypertension artérielle est observée chez 50% des cas, l'IMC moyen chez nos hémodialysés chroniques est de 24,21 ±3,22kg/m2, l'IMC, 3 de nos patients avaient une cardiopathie. La durée moyenne d'hémodialyse est de 69,3 ± 11 mois, 7 de nos patients avaient un rythme de 2 séances par semaine, tandis que les 5 restants étaient dialysés à raison de 3 fois par semaine, le KT/V moyen est de 1,40.

**Tableau 1:** Caractéristiques démographiques cliniques et dialytiques de nos patients diabétiques hémodialysé chronique

Caractéristiques	Résultats
Nombre de patients n(%)	12/94 (12,9%)
Age moyen (années)	53,2±5,6
Sexe (H/F)	7/5
Hypertension artérielle	50%
Index de masse corporelle moyen (Kg/m2)	24,21±3,22
Nombre de séances par semaine	2-3 séances/semaine
Durée moyenne de séances	4 heures
Débit de la pompe sanguine moyen	280ml/min
KT/V moyen	1,45

Sur le plan biologique 10(83%) patients étaient anémiques, avec une hémoglobine moyenne à 8,9 + /- 1,9. 7(58%) des patients avaient une hyperparathyroidie secondaire avec une PTH 1-84 moyenne de 508+-360 pg/ml, 3(25%) patients avaient une hypoparathyroidie, les paramètres biologiques des patients sont résumés dans le Tableau 2.

**Tableau 2:** Caractéristiques biologiques de nos patients diabétiques hémodialysé chronique

Paramètres biologiques sanguins	Moyenne ± Ecart-type (ET)
Hémoglobine (g/dl)	8,9± 1,9
PTH1-84 (pg/l)	508± 360
Calcémie (mg/l)	85,06±14,32
Phosphorémie (mgl)	61,10 ±12,34
25 OH Vitamine D	22,9 ± 8,2
Phosphatases alcalines (UI/l)	86,2 ±16
Cholestérol	2,11 ± 0,9
Triglycérides	1,98 ± 0,74

Sur le plan thérapeutique, 50% des patients recevait un traitement antihypertenseur, 7 patients étaient sous ASE. L'alfacalcidiol était prescrite chez 58% des patients, le traitement chélateur de phosphore était à base de calcium dans 58% des cas à une dose moyenne de 1,04g/j,

### Caractéristiques podologiques de nos patients diabétiques HDC

Tous nos patients diabétiques présentent au moins un trouble podologique. Aucun des patients n'a bénéficié de pontages ni angioplastie du membre inférieur alors que 8% ont subi des amputations. Le périmètre de marche est limité chez 75% des patients. 16,6% < 50 m, 16,6% < 100 m, 25% < 250 m, 16,6% ne marchent pas. Parmi, 58,3% présentent des troubles de la statique. 16,6% présentent des troubles trophiques. Quarante-cinq pour cent ont une neuropathie dépistée au test au brin de coton. Huit pour cent des patients portent des chaussures adaptées. Vingt-cinq pour cent des patients ont des plaies diverses (Tableau 3).

**Tableau 3:** Résume les caractéristiques podologiques de nos patients

Caractéristiques podologiques	Résultats (%)
Antécédents d'amputations du membre inférieur	8%
Antécédents de pontage ou angioplastie du membre inférieur	0%
Limitation du périmètre de marche	75%
<250m	16,6%
<100m	16,6%
<50m	16,6%
Ne marchent pas	25%
Troubles trophiques	16,6%
Troubles de la statique	58,3%
Neuropathie périphérique (Test au brin de coton)	45%
Plaies diverses	25%
Chaussures adaptées	8%

## Discussion

Dans notre série, 12,9% des patients HDC sont diabétiques et présentent des troubles podologiques, aucun de nos patients n'a bénéficié de pontages ni angioplastie du membre inférieur alors que 8% ont subi des amputations. Le périmètre de marche est limité chez 75% des patients. 16,6% < 50 m, 16,6% < 100 m, 25% < 250 m, 16,6% ne marchent pas. Parmi, 58,3% présentent des troubles de la statique. 16,6% présentent des troubles trophiques. Quarante-cinq pour cent ont une neuropathie dépistée au test au brin de coton. Huit pour cent des patients portent des chaussures adaptées. Vingt-cinq pour cent des patients ont des plaies diverses.

En raison de l'allure épidémiologique que prend le diabète sucré dans le monde entier, la prévalence de ses complications est amenée à s'accroitre significativement [6]. Le pied diabétique regroupe l'ensemble des affections atteignant le pied, représente un véritable problème de santé publique [9].

Dans les pays occidentalisés, chaque année deux patients diabétiques sur cent souffrent d'une ulcération de leur pied. Les chiffres concernant les amputations des membres inférieurs sont très variables: ainsi, l'incidence allant de moins de 1 ‰ en dans la région de Madrid ou au Japon pour dépasser 20 ‰ dans certaines tribus indiennes d'Amérique du Nord. En France, elle est estimée aux environ de 2 ‰ [8], ceci dit, le risque d'ulcération du pied et d'amputation du membre inférieur est beaucoup plus élevée dans la population diabétique: près de 25% des diabétiques présenteront un ulcère du pied au cours de leur vie [10].

Le patient dialysé diabétique est un patient à haut risque cardiovasculaire élevé avec la survenue fréquente d'artérite expliquant la nécrose tissulaire du pied diabétique [11]. La fréquence élevée des troubles podologiques chez le diabétique hémodialysé chronique est expliquée par l'augmentation de l'incidence de ces patients en hémodialyse chronique [11–13].

Les ulcérations podologiques représentent un facteur de surmortalité dans les populations diabétiques avec un risque de décès multiplié par 2,4 par rapport à un diabétique indemne de plaie [14]. L'infection complique l’évolution d'une plaie chronique 1 fois sur 4, en alourdit considérablement la prise en charge et augmente le risque d'amputation lorsqu'elle est associée à une artérite des membres inférieurs et / ou une ostéite sous-jacente [15], elle représente la cause de 1/4 des hospitalisations des patients diabétiques [16].

Les troubles trophiques du pied chez le diabétique sont la conséquence de plusieurs mécanismes physiopathologiques. Les connaître est essentiel afin d'en réduire l'incidence et d'en assurer une prise en charge précoce pour réduire le risque d'amputation. Trois complications du diabète sont principalement en cause: la neuropathie, l'artériopathie et l'infection. La neuropathie périphérique, sensitivomotrice et autonome, est fréquente et représente la principale complication à l'origine des lésions du pied diabétique, avec perte d'alerte douloureuse, déformations du pied, hyperappui et sécheresse cutanée. L'artériopathie est un facteur d'aggravation très important responsable de retard de cicatrisation et de gangrène à l'origine fréquente d'amputation [17].

Le pied est une cible privilégiée de ces complications du fait des zones d'hyperpression qu'il subit, de la prédominance distale des atteintes neuropathique et artériopathique et de l'atmosphère confinée, source de macération et de fragilité cutanée. Le diabète lui-même peut favoriser le risque par son ancienneté, son mauvais équilibre et son retentissement visuel. Enfin, certaines situations psychosociales, comme un syndrome dépressif, une hygiène défectueuse peuvent avoir un impact sur l'apparition des lésions du pied [18].

Le dépistage des patients à risque lésionnel et la mise en place de mesures de prévention se justifient par la fréquence et la gravité des amputations. La prévention reste un volet essentiel dans la prise en charge du pied diabétique, de nombreuses amputations chez ces patients peuvent être évitées grâce à une prise en charge multidisciplinaire préventive [13], qui s'appuie sur l'examen systématique des pieds et du chaussage qui est un point fondamental pour pallier l'absence de douleur et pour mettre en évidence des anomalies prélésionnelles. Il permet d'obtenir les éléments utiles pour la gradation des niveaux de risque en particulier l'examen au monofilament ou au brin de coton. Les indications d'orthèses plantaires et de certaines chaussures thérapeutiques sont fréquentes. Enfin, les patients les plus à risque doivent faire l'objet d'un suivi spécialisé auprès d'un pédicure podologue ou d'une structure spécialisée. Dans notre centre d'hémodialyse le dépistage se fait essentiellement par l'interrogatoire du patient et par un examen simple visant à chercher les troubles trophiques, statiques, les signes de neuropathie périphérique, et les plaies, la plupart des patients n'utilisent pas des chaussures adaptés par manque de moyens et absence de couverture sociale étant donné qu'ils appartiennent à un niveau socio-économique défavorisé dans la plupart des cas.

## Conclusion

Les diabétiques en HDC représentent une population de patients fragiles qui nécessitent une prise en charge multidisciplinaire visant d'abord la prévention, le diagnostic précoce des ulcérations du pied diabétique, le traitement adapté aussi bien de l'infection que de l’équilibre glycémique, ceci ne peut être réalisé sans un suivi et un programme d’éducation thérapeutique adapté.
